# MicroRNA Expression Profile of Whole Blood Is Altered in Adenovirus-Infected Pneumonia Children

**DOI:** 10.1155/2018/2320640

**Published:** 2018-10-14

**Authors:** Feng Huang, Junsong Zhang, Diyuan Yang, Yuelan Zhang, Jinxiang Huang, Yaochang Yuan, Xuefeng Li, Gen Lu

**Affiliations:** ^1^Guangzhou Women and Children's Medical Center, Guangzhou Medical University, Guangzhou, 510120 Guangdong, China; ^2^Institute of Human Virology, Zhongshan School of Medicine, Sun Yat-sen University, Guangzhou, 510080 Guangdong, China; ^3^The Second Affiliated Hospital of Guangzhou Medical University, the State Key Laboratory of Respiratory Disease, Guangdong Provincial Key Laboratory of Allergy & Clinical Immunology, The Sixth Affiliated Hospital of Guangzhou Medical University, Qingyuan People's hospital, Sino-French Hoffmann Institute, School of Basic Medical Sciences, Guangzhou Medical University, Guangzhou, 511436 Guangdong, China

## Abstract

Human adenovirus (Adv) infection is responsible for most community-acquired pneumonia in infants and children, which results in significant morbidity and mortality in children every year. MicroRNAs (miRNAs) are associated with viral replication and host immune response. Knowing the miRNA expression profile will help understand the role of miRNAs in modulating the host response to adenovirus infection and possibly improve the diagnosis of adenovirus-infected pneumonia. In our study, total RNA extracted from whole blood of adenovirus-infected pneumonia children and healthy controls were analyzed by small RNA deep sequencing. Expression profiles of whole blood microRNAs were altered and distinctly different in adenovirus-infected children. The top 3 upregulated miRNA (hsa-miR-127-3p, hsa-miR-493-5p, and hsa-miR-409-3p) were identified in adenovirus-infected children and provided a clear distinction between infected and healthy individuals. Potential host target genes were predicated and validated by qRT-PCR to study the impact of microRNAs on the host genes. Most of the target genes were involved in the MAPK signaling pathway and innate immune response. These highly upregulated microRNAs may have crucial roles in Adv pathogenesis and are potential biomarkers for adenovirus-infected pneumonia.

## 1. Introduction

Human adenovirus (Adv) infection is responsible for most community-acquired pneumonia in infants and children [1, 2]. Adv causes infections for 5–10% of upper and lower respiratory tract infections in children, which results in pneumonia and nearly 1.3 million deaths of children every year [3, 4]. The fatality rates for untreated severe pneumonia or disseminated disease caused by Adv may even exceed to 50% [5, 6]. There are no efficacious antiviral drugs for Adv treatment until now. Also, the traditional diagnosis of Adv infection is limited. Therefore, to discover the interaction between the virus and its host will help us to find novel treatment and diagnosis for Adv infection.

Human Advs are nonenveloped double-stranded DNA viruses and belong to the Adenoviridae family [7, 8]. Human Advs are divided into seven subgroups including 53 serotypes based on immunologic and biological characteristics. Adv replicates efficiently in human cells and triggers an innate immune response such as inflammatory response in the host cells. Also, viral infection has proved to have a great impact on cellular small RNA expression and gene expression [9–11]. Adv infects the host cells through binding to different cellular receptors such as coxsackievirus and adenovirus receptor (CAR) [12]. During Adv DNA replication, host cellular proteins such as nuclear factor I and POU2F1 are used by Adv [13, 14]. In turn, the host will trigger an innate immune response against Adv infection. However, the details of Adv and host interaction still remains poorly defined.

Small RNAs are important regulators that modulate development, proliferation, differentiation, and apoptosis of organisms [15, 16]. Small RNAs include microRNA (miRNA), siRNA, tRFs, piRNA, and rasiRNAs, which regulate gene expression in a wide range of processes such as viral replication and host immune response. miRNAs are the most well-studied small RNAs during the latest decades. miRNAs are very important regulators that modulate transcriptome changes [17]. miRNAs regulate gene expression in a wide range of physiological and pathological processes such as in immune response and viral replication [18]. Although miRNAs have been examined in Adv type 3-infected human laryngeal epithelial cells and Adv type 2-infected human lung fibroblast cells [19, 20], there is no research about small RNA profiling in whole blood of Adv-infected pneumonia children. In our study, we sought to present the different miRNA profiles between Adv-infected pneumonia children and healthy controls, identify candidate diagnostic biomarkers for pneumonia with Adv infection in children, and examine the role of miRNAs in host defense response in Adv-infected children.

## 2. Materials and Methods

### 2.1. Patients

The whole blood samples used in the study were obtained from Guangzhou Women and Children's Medical Center. Children diagnosed with human Adv pneumonia were included in the study. The diagnosis of human Adv pneumonia was considered certain when it was associated with the following criteria: (1) lower respiratory and/or systemic symptoms, (2) lung infiltration on chest radiography or computed tomography (CT) scan, and (3) positive results for human Adv IgM antibody in sera and/or human Adv DNA by PCR in throat swabs and/or bronchoalveolar lavage (BAL) fluid. A total of 33 samples from patients and 33 samples from healthy volunteers were used in the study. The ages of all patients (male or female) and healthy volunteers (male or female) range from one year to three years. The study was approved by the Ethics Committee at Guangzhou Women and Children's Medical Center (number 2014121815), and written informed consent was obtained from all guardians.

### 2.2. RNA Extraction and Small RNA Sequencing

The blood samples of the patients and volunteers were collected in anticoagulant tubes. The total RNA was isolated using RiboPure™ Blood RNA Isolation Kit (Ambion, USA) according to the manufacturer's protocol. The extracted small RNAs were treated with DNase before deep sequencing. RNA concentration was determined using a NanoDrop ND1000 system (Thermo Fisher Scientific, South San Francisco, CA), and their integrity was verified using an Agilent 2100 Bioanalyzer (Agilent Technologies, USA). Small RNA deep sequencing was performed as previously described [21]. A total of 3 samples from patients and 3 samples from healthy volunteers were used for small RNA deep sequencing in the study. The clinical characteristics of the 3 patients and 3 healthy volunteers are summarized in Table 1.

### 2.3. Cell and Virus Culture

Human primary lung fibroblasts (IMR-90) and human 293T cells were grown in Dulbecco's modified Eagle's medium (DMEM) (HyClone) supplemented with 10% fetal bovine serum (Invitrogen), streptomycin, and penicillin (Invitrogen). All virus infections were carried out in serum-free medium for 1 h, followed by addition of saved complete medium.

### 2.4. Transfection

miRNA inhibitors (GenePharma Co., China) were transfected into IMR-90 cells with Lipofectamine RNAiMAX (Invitrogen) at final concentrations of 100 nM according to the manufacturer's protocol.

### 2.5. Virus Growth Assay

IMR-90 cells transfected with miRNA inhibitors were infected with HAdV5 (isolated from patients) at an MOI of 10 in serum-free medium. Virus titers were determined 72 h after infection by plaque assays performed on 293T cells [22, 23].

### 2.6. Quantitative Real-Time PCR (qRT-PCR) Analysis

A qRT-PCR experiment was performed using the Power SYBR Green PCR Master Mix. Each reaction was performed in a 10 *μ*L volume system containing 0.5 *μ*L of cDNA, 0.5 *μ*L of each primer, 5 *μ*L of Power SYBR Green PCR Master Mix, and 3.5 *μ*L of ddH_2_O. The reactions were incubated in a 96-well plate at 95°C for 10 minutes, followed by 39 cycles of 95°C for 15 seconds and 62°C for 1 minute. For miRNA quantitation, 10 ng of total RNA was reverse-transcribed using specific stem-loop primers. U6 was used as an endogenous control.

### 2.7. Data Analysis

miRNA cluster and family information from miRBase (miRBase 20, http://www.mirbase.org/) was used to annotate the cluster of miRNAs. miRNA target genes were predicted based on two software: miRDB software (http://mirdb.org/) and TargetScan software (http://www.targetscan.org/).

### 2.8. Statistical Analysis

Data were analyzed using GraphPad Prism 6.0 software (La Jolla, CA, USA). The two-tailed Student's *t*-test was used to determine the significance of statistical data between two experimental groups. Data were considered significant at ^∗^*P* < 0.05, ^∗∗^*P* < 0.01, and ^∗∗∗^*P* < 0.001.

## 3. Results

### 3.1. Different Expressions of MicroRNAs in Adv-Infected Children vs. Healthy Children

**Figure 1:**
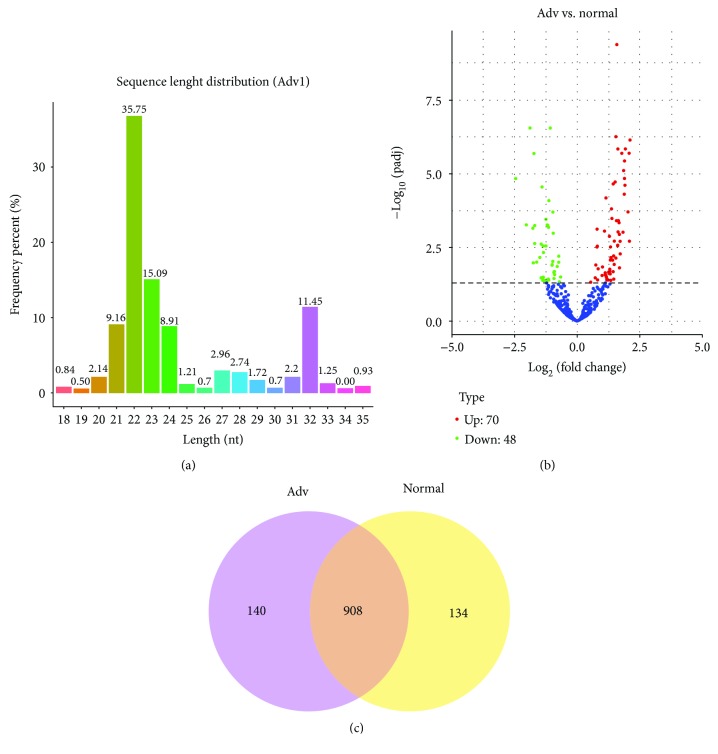
miRNA expression profile of whole blood from adenovirus-infected pneumonia children. (a) Deep sequencing shows the distribution of small RNAs of whole blood from adenovirus-infected pneumonia children. The miRNA peaks appear around 22 nt. (b) Volcano plot of differently expressed miRNAs between Adv-infected children and healthy controls. (c) Venn diagram of differently expressed miRNAs between Adv-infected children and healthy controls.

To study the impact of Adv infection on cellular small RNA expression in pneumonia children, deep sequencing of small RNAs was performed in our study. Accordingly, we found an apparent small RNA peak at 21–24 nt for miRNAs (Figure 1(a)). When we analyzed the differently expressed small RNAs, 118 miRNAs were found differently expressed in Adv-infected children vs. healthy children in a volcano plot (Figure 1(b)). We mapped the clean reads from each group to the known miRNA sequences and identified 908 miRNAs in Adv-infected children versus healthy controls (Figure 1(c)).

**Figure 2:**
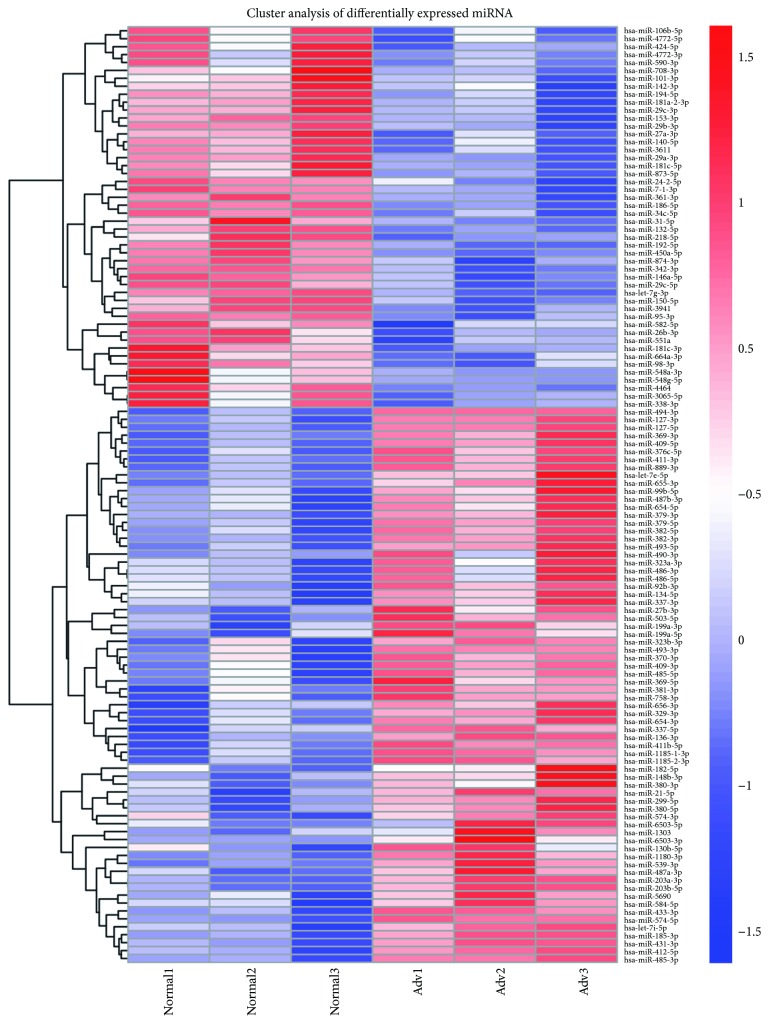
Hierarchical cluster analysis of differentially expressed miRNAs.

Furthermore, hierarchical cluster analysis of differentially expressed miRNA is shown in Figure 2. Specially, 77 differentially expressed miRNAs in the 6 samples passed our fold-change filter (log2 fold change > 1.0), among which 20 miRNAs that have high expression (reads up to 1000 in Adv samples) and showed significant different expressions were selected for further analysis (Table 2).

### 3.2. Validation of Differentially Expressed miRNAs

**Figure 3:**
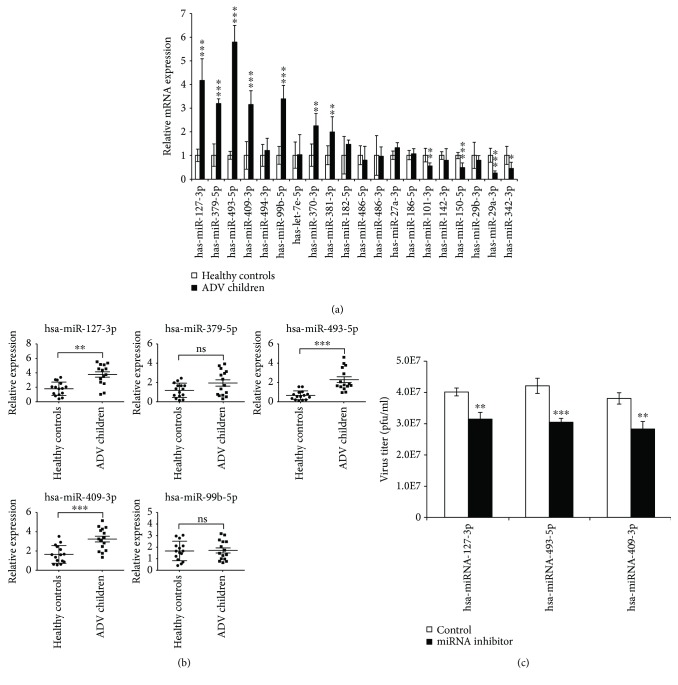
Quantification of miRNA expression levels by qRT-PCR. (a) Quantification of top 20 differently expressed miRNAs, including upregulated and downregulated miRNAs. Five samples from Adv-infected children and 5 from healthy controls were used in the experiment. (b) Quantification of top 5 upregulated miRNAs. 15 samples from Adv-infected children and 15 from healthy controls were used in the experiment. (c) The effect of miRNA inhibitors on the replication of Adv. After transfection with miRNA inhibitors, cells were infected with HAdV5 at an MOI of 10. Virus was harvested, and the titer was determined on 293T cells at the indicated time points. Data are shown as means ± SD from three independent experiments. ^∗∗^*P* < 0.01 and ^∗∗∗^*P* < 0.001 (Student *t* test).

To confirm the differential expression of miRNAs in Adv patients vs. healthy controls, we performed qRT-PCR assays in our study. The result showed that hsa-miR-127-3p, hsa-miR-379-5p, hsa-miR-493-5p, hsa-miR-409-3p, hsa-miR-99b-5p, hsa-miR-370-3p, and hsa-miR-381-3p were upregulated in whole blood samples from 5 Adv-infected children vs. 5 healthy controls (Figure 3(a)), while hsa-miR-101-3p, hsa-miR-150-5p, hsa-miR-29a-3p, and hsa-miR-342-3p were downregulated in whole blood samples from 5 Adv-infected children vs. 5 healthy controls (Figure 3(a)), which was comparable with our sequencing data. To identify candidate diagnostic miRNA biomarkers, we focused on the upregulated miRNAs. More samples (15 Adv-infected children vs. 15 healthy controls) were collected to verify the expression of the upregulated miRNAs (Figure 3(b)). From the result, we found that hsa-miR-127-3p, hsa-miR-493-5p, and hsa-miR-409-3p were significantly increased. Also, after depleting these miRNAs with miRNA inhibitors, the viral replications were significantly decreased (Figure 3(c)). These findings imply that our selected miRNAs may reflect the infection of Adv, and such miRNAs can likely serve as biomarker candidates for Adv-infected patients.

### 3.3. Predict Target Genes of Differentially Expressed miRNAs

**Figure 4:**
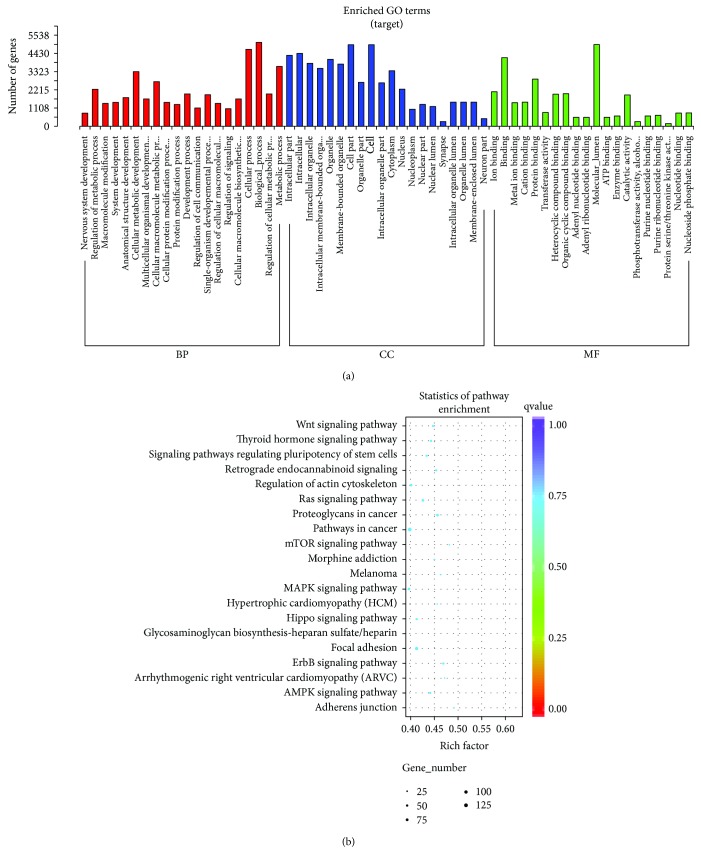
Go enrichment of predicted target genes. (a) The GO classification enrichment of target genes. (b) The KEGG pathway scatterplot of target genes.

To study the biological significance of miRNAs, we then predicted the computational target genes of miRNAs. We focused on the targets of verified miRNAs with distinct expression profiles. Go enrichment of the predicted target genes of the miRNAs showed that the target genes were mainly involved in cellular process and molecular function (Figure 4(a)). In particular, most of the target genes participated in the MAPK signaling pathway and Ras signaling pathway (Figure 4(b)). The top five predicted target genes of hsa-miR-127-3p, hsa-miR-493-5p, or hsa-miR-409-3p are listed in Table 3 by the highest miRNA target score from two miRNA predictive software.

**Figure 5:**
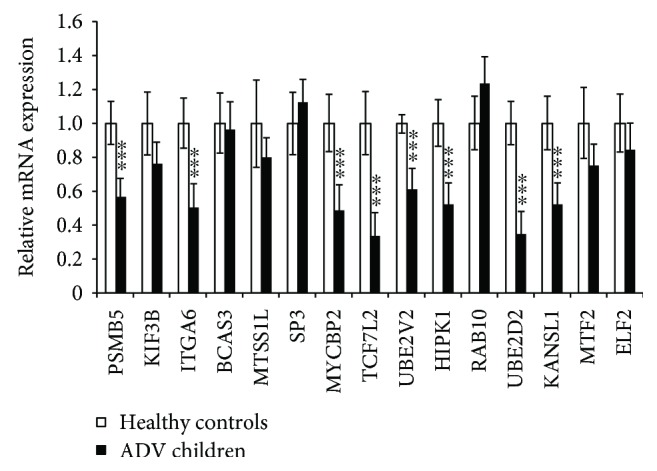
Validation of the expression of top 5 predicated target genes by qRT-PCR. Relative mRNA expression between 10 samples from Adv-infected children and 10 samples from healthy controls. Data are shown as means ± SD from three independent experiments. ^∗∗∗^*P* < 0.001 (Student *t* test).

The target genes were selected for further validation with qRT-PCR, and we found that the mRNA expression of 8 genes (PSMB5, ITGA6, MYCBP2, TCF7L2, UBE2V2, HIPK1, UBE2D2, and KANSL1) were downregulated in Adv-infected patients compared to healthy controls (Figure 5). Most of them are transcription factors or factors involved in the ubiquitin pathway. The downregulated mRNAs may indicate the mechanism of Adv infection and Adv-induced pneumonia.

## 4. Discussion

Human Advs are common causative pathogens of acute respiratory infections in children. The treatment of human Adv infections is limited because prospective, randomized therapeutic trials have not been done. Therefore, it is very important to discover the mechanism of Adv-infected pneumonia and search the biomarkers for Adv-infected pneumonia in children. The regulatory potential of miRNA is well defined, and the different profiles of miRNA expression are the result of diverse diseases including viral infections. In our study, we found that the miRNA profile in whole blood of Adv-infected children was different from that of healthy children. Blood samples of Adv-infected children reflect the associated pathology of Adv infection and thus provide a better understanding of the disease.

Profiling of miRNA expression from Adv-infected blood samples identified a cluster of 118 miRNAs significantly altered. The altered blood miRNA profile was similar to cells infected with Adv reported previously [19, 20], indicating that the different expressed miRNAs identified in our study could be taken as the candidate diagnostic biomarkers for pneumonia with Adv infection in children. Among these different expressed miRNAs, hsa-let-7e-5p was reported to involve in the replication of influenza infection [24]. hsa-miR-127-3p was reported to affect the Epstein-Barr virus-associated lymphoma through targeting the PTEN-AKT-mTOR pathway [25]. Altogether, these miRNAs play important roles in innate immune response or viral replication, thus affecting the outcome of the disease.

Through miRNA target gene GO analysis, we found that most target genes of different expressions of miRNAs were involved in the MAPK signaling pathway. The MAPK signaling pathway is activated by Toll-like receptors [26], which play important roles in innate immune response against viral infection. Also, the activation of the MAPK pathway will result in the activation of the NF-*κ*B signal pathway, which will stimulate the production of inflammatory cytokines and MMPs [27] and finally lead to pneumonia. Thus, the altered miRNA expression profile of whole blood from Adv-infected children partly reflected the mechanism of Adv-infected pneumonia.

With a developmental framework or disease process, miRNAs exhibit dynamic expression patterns. In our study, we characterized the miRNA expression profile of Adv-infected children using deep sequencing analysis and identified that many miRNAs were differently expressed in Adv-infected children when compared with healthy children. Those with the greatest differences were chosen for further verification. In particular, the expressions of hsa-miR-127-3p, hsa-miR-493-5p, and hsa-miR-409-3p from 20 Adv-infected children were significantly higher than those from 20 healthy controls, indicating that these miRNAs could be taken as good diagnostic biomarkers for Adv-infected pneumonia.

To further explore the possible molecular mechanisms of the differently expressed miRNAs in Adv-infected children, we predicted the possible target genes of hsa-miR-127-3p, hsa-miR-493-5p, and hsa-miR-409-3p and found that most of them are transcription factors or factors involved in the ubiquitin pathway. Especially, after verifying the predicted target genes with qRT-PCR, 8 genes (PSMB5, ITGA6, MYCBP2, TCF7L2, UBE2V2, HIPK1, UBE2D2, and KANSL1) were found significantly downregulated in samples from Adv-infected children. In particular, MYCBP2 is an E3 ubiquitin protein ligase [28, 29], which regulated the cAMP and mTOR signaling pathway. The mTOR signaling pathway plays a critical role in effector T cell function, the downregulation of which will result in impaired cell cytolysis and incapability of virus elimination. UBE2V2 is an ubiquitin-conjugating enzyme and is involved in the differentiation of monocytes, which can produce proinflammatory cytokines like MIP-1*β* [30, 31]. Thus, the downregulated UBE2V2 will result in abnormal differentiation of monocytes and more proinflammatory will be produced, which in turn aggravates pneumonia. UBE2D2 is essential for the activation of MAVS and RIG-I in response to viral infection [32–34]. The downregulation of UBE2D2 will result in abnormal activation of MAVS and RIG-I signal and virus clearance. These findings imply that the miRNA expression profile changed in Adv-infected children resulting in different transcriptome profiles, which reflects the mechanism of Adv replication and the formation Adv-infected pneumonia.

## 5. Conclusions

In summary, we identified the 3 most markedly differently expressed miRNAs in whole blood from Adv-infected children, which can be taken as biomarkers for Adv-infected pneumonia. Simultaneously, based on target gene prediction and qRT-PCR analysis, we found that genes MYCBP2, UBE2V2, and UBE2D2 may play important roles in viral replication and Adv-induced pneumonia. However, additional studies are necessary to clarify their roles in these processes, which will provide a physiological basis for the treatment of Adv-infected pneumonia.

## Figures and Tables

**Table 1:** Clinical features of healthy volunteers and patients.

Volunteer or patient	Age	Gender	Fever duration^a^ (days)	Laboratory characteristics^b^					Radiology^e^
				WBC (×10^9^/L) (5–12)	HsCRP (mg/L)(<5)	Specific IgM^c^ (IU/mL)	HAdV DNA^d^ (throat swabs/BALF)	LDH (U/L) (159–322)	Consolidation
V1	1y9m	male	−	8.6	−	−	−	−	−
V2	2y8m	female	−	9.0	−	−	−	−	−
V3	2y3m	male	−	5.7	−	−	−	−	−
A1	2y1m	male	13	10.2	26.5	+	+/+	919	+
A2	1y9m	female	16	8.3	51.14	+	+/+	812	+
A3	2y8m	female	22	2.8	10.98	+	+/+	948	+

^a^Fever duration from onset to normothermia. ^b^Data extracted from the first test for the children on admission. ^c^Specific IgM antibodies against HAdV pneumoniae were detected in 2 mL of acute phase (on admission) patient serum using a commercial ELISA kit. ^d^HAdV pneumonia DNA was detected by real-time PCR in throat swabs or bronchoalveolar lavage fluid (BALF). ^e^Judged by chest radiograph or CT scan in whole course of the patients.

**Table 2:** Top 20 different expressed miRNAs.

miRNA name	Reads in ADV	Reads in control	Log2 fold change	*P* value
has-miR-381-3p	9358	3507	1.17	0.00511
has-miR-486-5p	5480	2240	1.12	0.00434
has-miR-409-3p	4916	1223	1.61	0.00023
has-miR-486-3p	4745	1922	1.12	0.00402
has-miR-127-3p	3950	753	2.10	6.35*E* − 09
has-miR-182-5p	2396	870	1.17	0.00762
has-miR-99b-5p	2241	650	1.46	0.00066
has-miR-379-5p	1687	378	1.88	3.31*E* − 07
has-miR-370-3p	1682	617	1.18	0.00621
has-let-7e-5p	1239	429	1.33	0.00030
has-miR-493-5p	1089	237	1.87	1.63*E* − 06
has-miR-494-3p	1070	342	1.54	3.94*E* − 09
has-miR-101-3p	57,893	150,004	−1.12	0.00825
has-miR-142-3p	24,616	77,702	−1.25	0.00841
has-miR-150-5p	15,854	41,850	−1.25	0.00025
has-miR-29a-3p	6679	18,944	−1.40	9.01*E* − 07
has-miR-186-5p	6093	13,066	−1.07	1.49*E* − 09
has-miR-27a-3p	5558	11,953	−1.00	0.00170
has-miR-342-3p	3373	13,980	−1.87	1.37*E* − 09
has-miR-29b-3p	1339	3404	−1.25	1.60*E* − 05

**Table 3:** Top 5 predicted target genes.

miRNAs	Target	Gene description
hsa-miR-127-3p	PSMB5	Proteasome (prosome, macropain) subunit, beta type 5
	KIF3B	Kinesin family member 3B
	ITGA6	Integrin, alpha 6
	BCAS3	Breast carcinoma-amplified sequence 3
	MTSS1L	Metastasis suppressor 1-like

hsa-miR-493-5p	SP3	Sp3 transcription factor
	MYCBP2	MYC-binding protein 2, E3 ubiquitin protein ligase
	TCF7L2	Transcription factor 7-like 2 (T-cell specific, HMG box)
	UBE2V2	Ubiquitin-conjugating enzyme E2 variant 2
	HIPK1	Homeodomain-interacting protein kinase 1

hsa-miR-409-3p	RAB10	RAB10, member RAS oncogene family
	UBE2D2	Ubiquitin-conjugating enzyme E2D 2
	KANSL1	KAT8 regulatory NSL complex subunit 1
	MTF2	Metal response element binding transcription factor 2
	ELF2	E74-like factor 2 (ETS domain transcription factor)

## Data Availability

The data used to support the findings of this study are included within the article.
